# Electrical impedance-based tissue classification for bladder tumor differentiation

**DOI:** 10.1038/s41598-024-84844-9

**Published:** 2025-01-04

**Authors:** Carina Veil, Franziska Krauß, Bastian Amend, Falko Fend, Oliver Sawodny

**Affiliations:** 1 Institute for System Dynamics, University of Stuttgart, Waldburgstr. 19, 70563 Stuttgart, Germany; 2 Department of Urology, University Hospital Tübingen, Hoppe-Seyler-Str. 3, 72076 Tübingen, Germany; 3 Institute of Pathology and Neuropathology and Comprehensive Cancer Center, University Hospital Tübingen, Liebermeisterstr. 8, 72076 Tübingen, Germany

**Keywords:** Tissue differentiation, Bladder cancer, Electrical impedance, Data analysis, Feature extraction, Urology, Biomedical engineering, Surgical oncology, Scientific data

## Abstract

Including sensor information in medical interventions aims to support surgeons to decide on subsequent action steps by characterizing tissue intraoperatively. With bladder cancer, an important issue is tumor recurrence because of failure to remove the entire tumor. Impedance measurements can help to classify bladder tissue and give the surgeons an indication on how much tissue to remove. Over the years of research, it became obvious that electrical impedance spectroscopy is a very promising tool for tissue differentiation, but also a very sensitive one. While differentiation in preliminary studies shows great potential, challenges arise when transferring this concept to real, intraoperative conditions, mainly due to the influence of preoperative radiotherapy, possibly different tumor types, and mechanical tissue deformations due to peristalsis or unsteady contact force of the sensor. This work proposes a patient-based classification approach that evaluates the distance of an unknown measurement to a healthy reference of the same patient, essentially a relative classification of the difference in impedance that is robust against inter-individual differences and systematic errors. A diversified dataset covering multiple disturbance scenarios is recorded. Two alternatives to define features from the impedance data are investigated, namely using measurement points and model-based parameters. Based on the distance of the feature vector of a unknown measurement to a healthy reference, a Gaussian process classifier is trained. The approach achieves a high classification accuracy of up to 100% on noise-free impedance data recorded under controlled conditions. Even when the differentiation is more ambiguous due to external disturbances, the presented approach still achieves a classification accuracy of 80%. These results are a starting point to tackle intraoperative bladder tissue characterization and decrease the recurrence rate.

## Introduction

Bladder cancer prevalence is especially high for men in developed countries and mainly linked to tobacco consumption^1^. With a share of 95%, urothelial carcinomas are the most common carcinomas of the bladder. They grow towards the inside of the bladder and are mostly discovered in early, non-muscle invasive stages, allowing for a minimally invasive tumor removal through the urethra. In the endoscopic procedure, the surgeon removes cancerous tissue and the surrounding areas based on a visual inspection. Histopathological sections are then used to verify that the entire tumor has been removed^2^. Even though many advances in urological oncology have been made, such as photodynamic diagnostic to visualize the tumor cells, the bladder cancer recurrence rate due to leftover tumor cells is still very high^3^. Once the tumor is too advanced and invaded the muscle, the entire bladder needs to be removed, a step that surgeons want to avoid as it comes with a high risk surgery and a decrease in the quality of life. A promising research direction is to include sensors from multiple domains to provide intraoperative information about the physical properties of the tissue and help the surgeons identify the tumor and its margins to ensure full removal in the endoscopic procedure^4^. As a tumor undergoes biochemical and structural changes^5^, such as increased proliferation of cells, the tissue’s electrical behavior changes and a possible differentiation can be made through impedance measurements, which is especially promising giving the fact that electrocautery is already used for resection and possible synergies in the tools used arise from that.

Dielectric properties of biological tissue have been studied and measured continuously since the emergence of suitable measurement equipment^6–9^. Pathological changes in cancerous tissue affect cell structure, size, and molecular composition, and impedance measurements have already been successfully used for tumor detection in various applications. However, these tumor locations are often easily accessible from outside the body, for example skin cancer^10,11^, cervical carcinomas^12–14^, and oral cancer^15,16^. Or, the measurements take place during an open surgery, for example thyroid^17^, or breast^18–20^, with a working area of several centimeters. A difficulty for transurethral surgery lies in the small endoscopic working channel, which confines the sensor diameter to a maximum of 2 mm and comes with additional challenges; such as the mechanical deformation of the bladder or the unsteady contact force between sensor and tissue that strongly impact the measured impedance due to shifted fluid contents - resistivity increases up to 30% over time under ongoing mechanical tissue deformation^21^.

### Related work

#### Impedance sensors

Tissue impedance refers to the opposition that the tissue present to the flow of electrical current, particularly in the context of alternating current (AC). Like electrical impedance in circuits, tissue impedance is a complex quantity with both resistive and reactive components, and defined as the ratio

1 $$\begin{aligned} \underline{Z}(\omega ) = \frac{\underline{v}(\omega )}{\underline{i}(\omega )} = R(\omega ) +jX(\omega ) \end{aligned}$$

of alternating voltage $\underline{v}(\omega )$ and current $\underline{i}(\omega )$ of frequency $\omega$. Its real part is called resistance $R(\omega )$, and the imaginary part is referred to as reactance $X(\omega )$. As measured impedance depends both on tissue properties and the measurement setup, it is not always a suitable quantity to use for tissue differentiation. Normalizing impedance with a measurement setup specific geometry factor *k* yields the impedivity $\underline{z}(\omega )= \nicefrac {\underline{Z}(\omega )}{k} = \rho (\omega )+jx(\omega )$ with resistivity $\rho (\omega )$ and reactivity $x(\omega )$. It allows for the determination of material values such as conductivity and permittivity that are comparable to literature data and among different impedance sensors used^22^.

When trying to classify tumorous tissue based on impedance, the choice of the sensor plays an important role. As stinging needle-shaped electrodes in the tissue favors the spreading of tumor cells^23,24^, and the area is usually not accessible from both sides, sensors with needle electrodes or a transversal measurement setup are not suitable. For the detection of bladder cancer, impedance sensors should be designed such that they are placed on the surface of the tissue and measure the area underneath.

Tissue impedance is usually measured with either two (bipolar) or four (tetrapolar) electrodes. For bipolar sensors, the electrodes carry the current and measure the voltage at the same time, which makes their design easier but also renders the measurement susceptible for interference. Tetrapolar sensors use separate pairs of electrodes to carry the current and measure the voltage, which prevents the majority of contact effects^22,25^. They are mainly used for biomaterials and ionic conductors, namely applications where the electrodes are in contact with a material that exhibits contact effects. The sensors are usually designed with four circular, quadratically arranged electrodes and have been used for tissue measurements in various applications^26–28^. Approved medical devices exist, for example, for the detection of cervical cancer^29,30^. A novel tetrapolar impedance sensor design with ring electrodes for an improved and interference free electrical measurement, and an outer diameter of 2 mm, suitable for endoscopic bladder tumor removals, was presented in^31^ and extensively validated in our previous contributions.

#### Altered electrical properties of tumor

Changes in the electrical properties of tumorous tissue have already been reported in the early twentieth century^32^. Table 1 presents a selection of studies evaluating the potential of electrical impedance measurements to differentiate healthy and tumorous tissue. It is limited to bipolar or tetrapolar surface sensors that would be suitable for the application of bladder cancer. However, note that the majority of the sensors does not have a small enough diameter to fit through the urethra in an endoscopic setup. So far, the majority of the studies mentioned in Table 1 are carried out ex vivo, except at body parts that are easily accessible from the outside and do not require surgery, such as mouth, skin, or cervix.

**Table 1:** Selection of studies dealing with tissue differentiation using electrical impedance for different organs.

Tissue		Year	Tumor impedance^1^...	Source
Cervix	In vivo	2000–2020	Resistivity $\downarrow$ permittivity $\downarrow$	^13,29,30^
Thyroid	Ex vivo	2018, 2020	Resistivity $\uparrow$ permittivity $\uparrow$	^17,33^
Breast	Ex vivo	1998–2019	Resistivity $\downarrow$ permittivity $\uparrow$	^18,19,34–36^
Skin	In vivo	2000–2022	Impedance magnitude $\downarrow$ impedance phase $\downarrow$	^10,11,37–42^
Prostate	Ex vivo	2007–2012	Resistivity $\uparrow$ Permittivity $\downarrow$	^43,44^
Bladder	Ex vivo, in vivo	2002	Resistivity $\uparrow$	^27,45,46^

^1^ Not all papers provided exact indications and values, some provide the differentiation based on model parameters. The following are assessments based on the provided data in each publication

Table 1 shows that there is no clear picture if impedance of tumorous tissue is increased or decreased in general. Biochemical reasons in tumor might suggest decreased impedance because of increased water and salt contents inside and outside the cells, as well as looser cell–cell connections^5,47,48^, but from literature, it seems that the direction of the change depends on the tissue type and histological grading: for example, decreased impedance can be observed in breast cancer, whereas increased impedance was observed in prostate tumors^18,43^. One reason for this is that in prostate cancer tumor growth reduces the well conducting extracellular space, whereas in breast cancer poorly conducting adipose tissue is replaced by better conducting tumor cells.

#### Tissue classification

There exist several proofs of concept in the literature using machine learning algorithms to classify tissue with neural networks (NN) or support vector machines (SVM). They deliver promising results for breast^19,49,50^, cervical^51^, skin^42,52^, or lung cancer^53^.

More precisely, ^19,50^ are based on the same breast tissue dataset^34^ with 120 measurements from 64 patients and achieve 92% to 97% accuracy with linear discriminant analysis and SVMs, respectively.^49^ achieves accuracies between 83% and 94% with NNs, on 70 training samples and 36 test samples. For skin, ^52^, presents an accuracy of 69% with SVMs and 81% with a naive Bayes classifier for the detection of melanoma on 21 patients, whereas^42^ achieves a sensitivity of 98% for the detection of melanoma, with a total dataset of 751 samples from 37 patients. For the latter, however, the classification algorithm is not specified. Last but not least, lung cancer was classified with an accuracy of 95% on average for a multitude of classifiers such as SVM, linear discriminant analysis, and *k* nearest neighbors.

A first approach with different classifiers to differentiate between impedance measurements on healthy and tumorous bladder tissue was presented in our previous work^54^. On a reduced, uniform dataset with tissue samples from four patients without any therapy and the same tumor type, we investigated the most suitable features for classification and achieved accuracies of up to 94% for different classifiers used. However, neither our previous approach nor one of the mentioned approaches from literature take into account the potential effect of preoperative therapy that might change the electrical properties of the tissue, or the occurrence of different tumor types in one bladder. A promising research direction for this challenges is a reference-based approach as proposed in^38^ where the measurement to classify is subtracted from a healthy reference measurement for studying the changes of impedance in skin cancer.

### Problem statement

A broad and well-known overview of electrical tissue properties is given in^55–57^, containing an extensive literature survey and measurements on multiple tissues. The authors themselves stated that they encountered “wide variations in the conductivity values obtained for the same tissue in various studies”. This underlines that electrical measurements are very prone to the measurement setup but also to factors such as temperature, deformations, time of death, surface fluids, or dehydration^22,58,59^. Furthermore, tissue is anisotropic and heterogeneous and can vary for different individuals depending on their age or general health^60^.

These factors influencing tissue impedance complicate tissue differentiation. We discussed in our earlier work the effect of mechanical deformations, tissue relaxation, and tremor in more detail^21,61,62^, which are especially present in an intraoperative setup where instruments are held by hand and the organs are deformed by peristalsis or pulse. More precisely, compressing or stretching the tissue induces the flow of free fluids to adjacent structures and leads to an increase in impedance. On top of that, inter-individual differences exist, such as sex or age^63^. Another important factor that has not been taken into account so far is the influence of preoperative therapy, e. g. radiotherapy prior to the surgery. These therapies change the physical properties of both the tumorous and the adjacent healthy tissue, and their impact is already challenging for state-of-the-art tissue differentiation methods^64,65^.

All these influencing factors make it difficult to break down, for example, urinary bladder conductivity to one frequency-dependent curve which would be considered “healthy”, making a global classification across different patients difficult: while the distinction in impedance measurements from healthy and tumorous spots of one patient usually gives a clear indication, tumorous measurements of one patient might overlap with healthy measurements of another patient, even more when different deformations or preoperative therapies play a role. New intraoperative tissue classification techniques need to take these limitations into account in order to enable robust and reliable tissue classification in real-time.

### Contribution and structure

The aim of this work is to develop an impedance-based bladder tissue classification concept able to deal with the mentioned external disturbances, such as deformation, preoperative radiotherapy, and inter-individual differences, making it suitable to be applied in endoscopic surgery scenarios. Our previous works (as well as works from other researchers, e.g.^27,55–57^), have shown us the sheer impossibility to produce consistent impedance values due to the multitude of disturbing factors that can occur. How would we be able to account for surgery decisions made based on a single impedance value without having a technical specialist supporting the surgeons decision in surgery, making sure the measurement is valid? Hence, we do not claim to provide exact electrical tissue values for healthy and tumorous bladder in this work, but to propose a method that allows for a differentiation even under the influence of such systematic errors, giving an outlook for robustness in real-life applications of impedance spectroscopy in surgery.

To do so, we adopt a reference-based approach, where an unknown impedance measurement is classified by evaluating its distance to a measurement taken at a healthy part of the bladder of the same patient, inspired by the works of^38^ for skin cancer, whose results were, however, also limited in terms of data availability, stating themselves that “a study on a dataset of this size is not conclusive”^38^. Further, they only considered subtracting measurement points, whereas we also investigate the subtraction of model parameters. The preliminary findings of^38^ are sufficiently promising to investigate this approach further and determine if it can deal with the special difficulties arising in bladder tissue differentiation.

In this work, for the classification of said difference, which we can interpret as a distance in a high-dimensional space, we use a Gaussian process classifier (GPC) with the binary output *healthy* and *tumorous*: Simply speaking, if the distance between both measurements is small, the unknown measurement is presumably also healthy. A large distance indicates that the unknown measurement is tumorous. A GPC has only few parameters to be optimized and can thus lead to a good generalization even on smaller datasets^66^. The GPCs predictive performance is similar to more commonly used tools like SVMs and NNs. However, if the probabilistic output is of interest, the GPC outperforms other kernel algorithms, because it naturally incorporates the confidence interval in its predictions^67^ - very promising for medical applications in general. A measurement being classified as 55% tumorous would more likely lead to reinvestigation than one classified as 95% tumorous. To train, validate and test the algorithm, a diverse dataset covering all above mentioned scenarios is created: different tumor types, preoperative therapy and varying mechanical measurement conditions. The data is obtained from six patients, with up to several hundred measurements per patient. Preliminary results for this work concerning feature extraction and selection on a smaller and uniform dataset without external disturbance have been published in^54^.

*Structure* The remainder of this work is structured as follows. In “Data acquisition” section presents the data acquisition in terms of experimental setup and data diversification. In “Feature definition” section, different features types are presented and extracted from the impedance measurements. Subsequently, the resulting feature vectors are evaluated by implementing a patient-based distance measure to a healthy reference measurement in “Reference-based feature evaluation” section. The classification step with a GPC and the results of the approach are topic of “Gaussian process classification” section. The work is wrapped up with a discussion and conclusion in “Discussion” and “Conclusion” sections.

## Data acquisition

### Impedance measurement

**Fig. 1:**
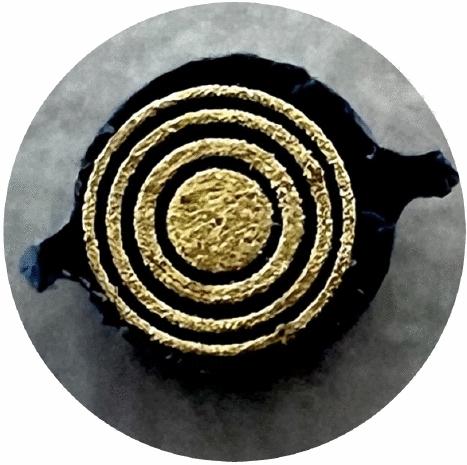
Impedance sensor tip that is in contact with the tissue. It has a diameter of 2 mm and was fabricated using laser-direct structuring. With the tetrapolar measurement setup, current is injected through the central electrode and lead out through the outer ring. The voltage measurement takes place through the two ring electrodes in between.

The impedance sensor used has a diameter of 2 mm and was extensively presented and validated in^31^. The sensor tip is depicted in Fig. 1: The contact area to the tissue has three ring-shaped electrodes around one central, circular electrode. A small, non-pathological current $\underline{i}_\textrm{cc}(\omega )$ (cf. standards for medical equipment^68^, DC current amplitude < 10 mA) is injected through the central electrode and the outer ring acts as current sink. The voltage measurement $\underline{v}_\textrm{vm}(\omega )$ is carried out in between the two inner rings, i. e.

2 $$\begin{aligned} \underline{Z}(\omega ) = \frac{\underline{v}_\textrm{vm}(\omega )}{\underline{i}_\textrm{cc}(\omega )}. \end{aligned}$$

The subscripts *vm* and *cc* refer to the terminology of the *voltage-measuring* and *current-carrying* electrode pair present in tetrapolar impedance measurement setups with four electrodes. The geometry factor of the sensor was determined to be 145 $\hbox {m}^{-1}$ through both simulations and measurements on salines with known conductivities^31^; it allows for the transformation of the measured impedance values to tissue specific characteristics such as conductivity and permittivity. Thanks to the ring-shaped geometry, the electrical field created from the central electrode to the outer ring is symmetrical underneath the sensor surface. Additionally, the outer ring being the current sink has the effect of a shield electrode. We could show in simulations that this geometry allows for an improved tumor detection especially if not the entirety of the tumor is covered by the sensor. The sensing penetration depth is approximately 1 mm which enables the sensing of superficial tumors (as they occur in the bladder)^69^.

The tetrapolar impedance measurement itself is carried out with the *Zurich Instruments Multi Frequency Impedance Analyzer*. The device uses lock-in amplification for the impedance measurement, hence, instead of specifying a current, the user specifies the magnitude of the voltage signal. By setting the voltage RMS to 0.5 V, we can estimate that the resulting current is below the threshold of < 10 mA as long as the resistivity of the tissue is above 0.34 $\Omega \hbox {m}^{-1}$. This is the case for this study (cf. Fig. 4), especially considering that 10 mA is the DC threshold and the AC current threshold increases as a function of frequency.

The frequency range for the measurement is set to 1 kHz to 1 MHz. Tissue exhibits different characteristics in different frequency domains. The conductivity is mainly determined by the intra- and extracellular fluid: the more fluid is present, the higher the conductivity^70^. Schwan identified a decrease of the permittivity in three major steps, the so-called dispersions, which correlate with the polarizability of different intra- and extracellular components in the respective frequency domains^71^. Due to the fact that conductivity is inferred from the real part of the impedance, whereas permittivity relates to its imaginary part, any decrease in permittivity with frequency is accompanied by an increase in conductivity according to the Kramers-Kronig relations^8^. These mathematical relations connect the real and imaginary parts in causal, physical systems^72^. Hence, the three characteristic dispersion regions, named $\alpha$, $\beta$, and $\gamma$ also correspond to relevant decreases in impedance^7,9,71,73^. From a biological point of view, the $\beta$-dispersion region (kHz to a few MHz) is most insightful for cancer growth, because the current starts penetrating the cells instead of only flowing around and impedance measurements can detect changes in intracellular components and fluids as well as membrane capacity. Measurements starting at a frequency of 1 kHz are sufficient and ensure no mistaking contact issues for tissue characteristics^31^.

### Diversification of patient data

The measurements presented in this work have been taken in the Department of Urology, University Hospital Tübingen, Germany. The tissue resulted from patients undergoing radical cystectomy, which made it possible to get both healthy and tumorous measurements from the same bladder. Overall, the data from six patients, in the following called P1 to P6, was collected. All methods were performed in accordance with the relevant guidelines and regulations.^1^ As measurements are only possible ex vivo, we could only include patients whose bladder cancer was advanced enough to make a surgical removal of the entire bladder necessary. The tumors are highly heterogeneous, of different grading and staging, and include

- highly undifferentiated urothelial carcinomas (P2, P6)
- urothelial carcinoma with squamous differentiation (P3)
- sarcomatoid urothelial carcinoma (P1),
- mucinous adenocarcinoma (P4)
- muscle invasive urothelial carcinoma (P5)

Additionally, patients P1, P2, P3, P4 underwent preoperative radiotherapy to shrink the tumor before the bladder removal. More detailed information is provided in Table S1 of the Appendix.

The low sample size with high diversity in the data set raises the question whether generalizing across these different tumor types is possible, especially considering the inclusion of a mucinous adenocarcinoma. Across all histologically defined subtypes of urothelial carcinoma, the tumor mass is consistently characterized by the proliferation of epithelial cells surrounded by variably desmoplastic stroma and in part immune cells. Although the growth pattern and the stromal composition differ between adenocarcinoma and urothelial carcinoma, the destruction of normal compartments and proliferative and inflammatory activity separate both from conventional urothelial morphology. The composition of the tumor environment and the hallmark features of malignant tumors may universally help in tumor mass identification through impedance measurements. Moreover, as normal tissue is used as reference to quantify the tumor impedance, we show in “Resulting feature space for different tumor types” section that our features capture the characteristics of the tumor growth itself, making the approach suitable for different tumor types.

### Diversification of mechanical conditions

**Fig. 2:**
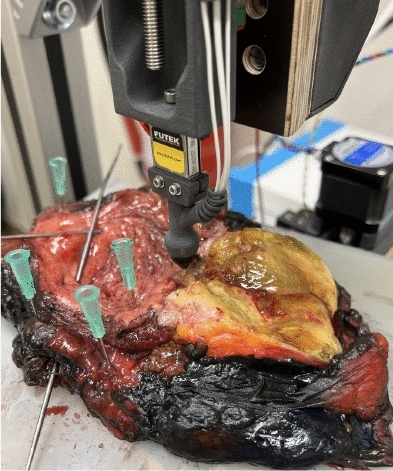
Impedance measurements on a human bladder with an automated test rig. The impedance sensor tip from Fig. 1 is mounted underneath a force sensor and a linear axis, which allows for the automatic control of the sensor-tissue contact force.

**Fig. 3:**
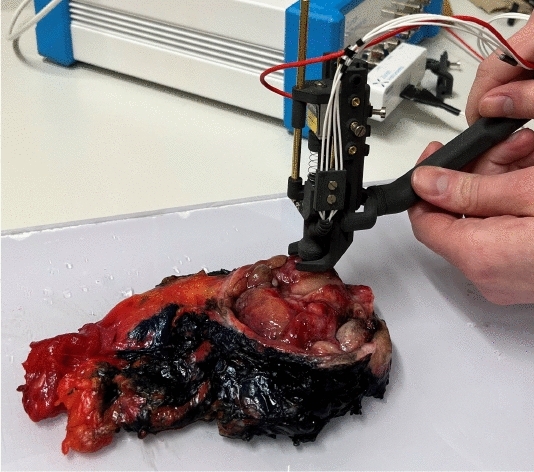
Impedance measurements on a human bladder with a handheld construction around the impedance sensor tip from Fig. 1 to catch the effects of unsteady measurement conditions.

To reenact different intraoperative scenarios with multiple deformations, the sensor tip was integrated in two different test setups:

- A test rig where the tip is mounted on a linear axis underneath a force sensor to allow for the automatic control of the sensor-tissue contact force and avoid movements during the impedance measurement is shown in Fig.  2.
- A handheld construction to carry out impedance measurements by hand to catch the effects of unsteady contact force and tremor is shown in Fig.  3.

The majority of the measurements (P1-P5) is carried out with the test rig and different predefined contact forces between 0.5 N and 1.5 N to provide as much noise-free data as possible. This force range was determined to be representative of the force expected with a handheld impedance sensor in previous works^62^, and chosen in order to make both the measurements with the test rig and the handheld construction comparable in terms of forces. A smaller portion of the measurements (P6) is carried out with the handheld device: Such disturbed measurements would be a challenge for any classification algorithm and can be used to investigate the performance of the proposed approach for measurements suffering from disturbances.

### Experimental procedure

The entire removed bladder was provided for measurements directly after surgery for the duration of 1 h before being analyzed in pathology. The tissue was kept moisturized with saline and all experiments took place in ambient temperature. Pathologist and urologist stood close by to show healthy, tumorous and suspicious spots in the bladder and systematically mark them in order to provide histopathological results for the measured spots afterwards. In total, 793 measurements from six patients were taken (cf. Table 2).

**Table 2:** Amount of impedance measurements taken.

Patient	Healthy	Tumorous
1	105	110
2	45	180
3	90	45
4	16	20
5	36	90
6	16	14

### Impedance measurement results

**Fig. 4:**
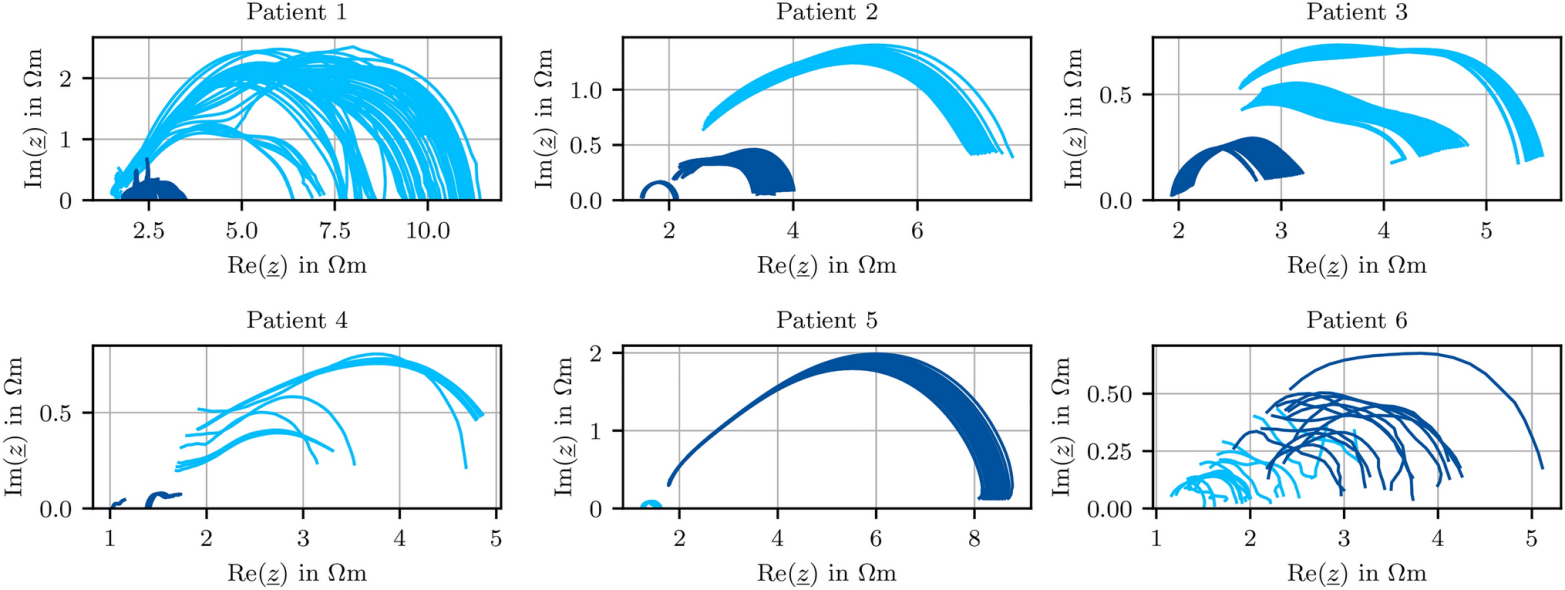
Complex plane diagrams of healthy () and tumorous () impedivity measurements from all patients in the frequency range 1 kHz to 1 MHz.

All measurements are depicted in form of complex plane plots in Fig. 4. Preoperative therapy in P1-P4 increases the impedance of the surrounding, healthy tissue drastically relative to the literature values and the tumorous tissue measurements. Unsteady contact force leads to unsmooth measurement curves in P6. Especially due to the influence of therapy, and as these changes differ in terms of absolute values among these patients, a global differentiation is difficult, as healthy measurements of one patient overlap with tumorous measurements of another patient.

In^46^, the resistivity of healthy and tumorous bladder tissue of 16 patients was published. At a frequency of 2 kHz, the mean value of healthy bladder resistivity was (3.3 ± 1.6) $\Omega \hbox {m}$, and the mean value of tumor resistivity was (6.5 ± 3.5) $\Omega \hbox {m}$. Literature suggests a mean value of 3.14 $\Omega \hbox {m}$ for healthy urinary bladder wall for frequencies below 1 MHz, a quite broad spectrum^74^. Within our work, the values differ: healthy resistivity was found to be (6.4 ± 4.3) $\Omega \hbox {m}$, and tumourous resistivity (3.8 ± 1.6) $\Omega \hbox {m}$, including all examined bladders with different tumor types and therapies involved. Having measured on tissue without the impact of preoperative therapy before, the authors assume that this is mainly explained by the impact of preoperative therapy. The finding that mainly healthy tissue is impacted by said therapy and might exhibit similar resistivity values than tumorous resistivities of other studies such as^46^ emphasises the multitude of difficulties arising in impedance-based tissue classification and the necessity of a reference-based approach as suggested in this work and allow for a robust differentiation in real intraoperative conditions.

## Feature definition

Each of the 793 measurements is represented by a complex vector of dimension 50, i. e. the impedance measurement points at 50 logarithmically spaced frequencies in the predefined kHz-range. Before starting to train a classification algorithm, adequate features need to be chosen from the measurements. More details on how to extract and select the most influential features from bladder tissue measurements can be found in our previous work^54^. In the scope of this work, both a classification based on a reduced measurement vector and based on model parameters are pursued and introduced in the following subsections. Later, in “Gaussian process classification” section, both approaches will be compared to evaluate which type of features yields a better classification accuracy with a diversified dataset and a reference-based classification approach.

From now on, let $\varvec{X}\in \mathbb {R}^{n_\text {s} \times n_\text {f}}$ be the input data for a machine learning algorithm for classification. The number of features is denoted by $n_\text {f}$ and the number of samples by $n_\text {s}$.

### Using a reduced measurement vector

When the features are defined by the measurement points, the amount of frequencies within the impedance measurement vector determines the quantity $n_\text {f}$. As real and imaginary parts of these measurement points are considered separately to avoid complex input data, using the entire vector would results in a high-dimensional feature space with $n_\text {f}=100$. To favor faster convergence and avoid overfitting with the relatively small dataset, the five most promising measurement points are determined based on a principal component analysis (PCA). This does not only reduce the dimensionality of the feature space but also shortens the measurement duration for a future application.

**Fig. 5:**
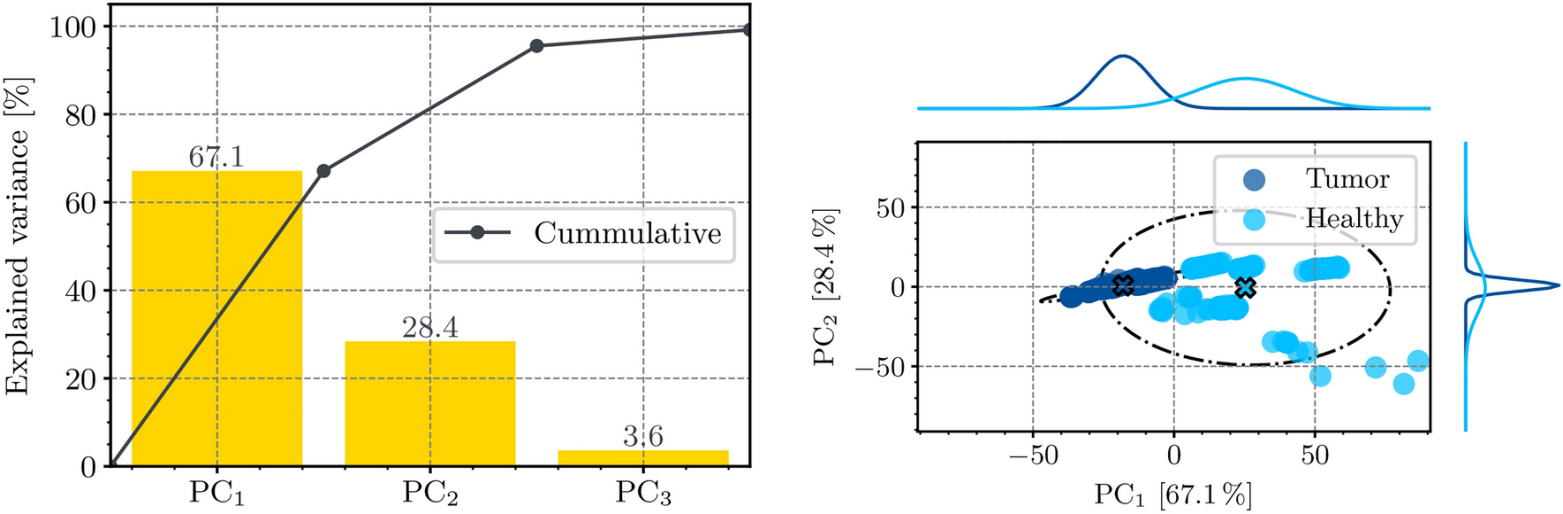
Scree plot (left) and scores plot (right) of the PCA. The score plot shows the percentage of explained variance for each of the first three principal components. The scores plot depicts the data in the space of the first two principal components. The ellipsoids of the scores plot correspond to a confidence interval of three standard deviations around the center of the scattering. They are projected onto the corresponding axes. The ellipsoid of the tumor data is very small and covered by the data points.

In order to allow for a high resolution on the frequency axis, the PCA is based on 50 logarithmically spaced values between 1 kHz and 1 MHz. The PCA on a subset of the training data (P1-P3) is depicted in Fig. 5 in the form of a scree plot and scores plot. The scree plot shows the sorted principal components based on their explained variance. As the first two principal components account for 95.5% of the variance, considering them is sufficient to analyze the variance of the data.

For the frequency selection itself, a cross-validation procedure is applied. The three training patients correspond to three healthy and three tumorous samples, and this data is combined into nine different subsets, which each consists of a unique combination of four of the six tissue samples. For each subset, a PCA is carried out for real and imaginary parts separately and the two first principal components are summed up along the feature axis such that each frequency is assigned an absolute value. For each cross-validation step, the ten frequencies with the highest values are selected.

**Fig. 6:**
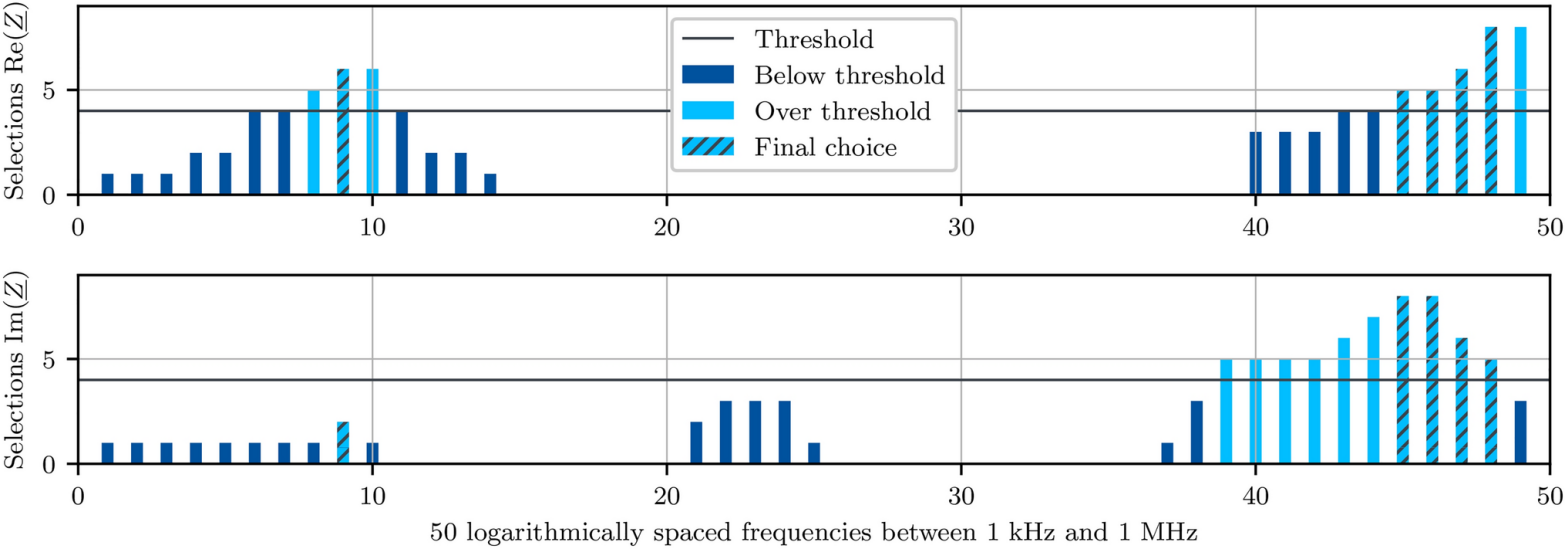
Number of selections for each frequency throughout the nine runs with different subsets of the training data. For every data combination, a PCA is carried out separately for the real and imaginary parts and the ten frequencies with the highest influence on the principal components are chosen.

Figure 6 shows how often each frequency has been selected throughout the nine steps. High frequencies seem important for both real and imaginary parts, whereas lower frequencies only show variance in the real part. All frequencies that have been selected in more than four of the nine steps are used as features. Additionally, one frequency of the lower half of the spectrum is chosen to account for the information in the real part. This results in the measurement points at the frequencies 3.55 kHz, 568.99 kHz, 655.13 kHz, 754.31 kHz, and 868.51 kHz as final features. In doing so, the dimension of the input data is reduced to five frequencies, resulting in input data $\varvec{X}\in \mathbb {R}^{n_\text {s}\times 10}$.

### Using model-based parameters

Alternatively, instead of a reduced measurement vector, a model-based analysis of the impedance measurement can allow for the combination of information in fewer parameters than the original measurement and visualize additional dependencies. A commonly used model to represent the electric properties of tissue is the Cole model

3 $$\begin{aligned} \underline{z}_\text {Cole}(\omega ) = \rho _{\infty }+ \frac{\rho _0-\rho _{\infty }}{1 +\left( j\omega \tau \right) ^\alpha }, \end{aligned}$$

which describes the (normalized) tissue impedance based on a low frequency resistivity $\rho _0$ in $\Omega \hbox {m}$, the a frequency resistivity $\rho _{\infty }$ in $\Omega \hbox {m}$, the characteristic frequency $\nicefrac {1}{\tau }$ in $\hbox {s}^{-1}$, and a dimensionless parameter $\alpha \in (0,1]$^73,75^. For each measurement, these parameters $\underline{p}=[\rho _0, \rho _{\infty }, \tau , \alpha ]$ are determined using a least-squares fitting algorithm, i.e.

4 $$\begin{aligned} \min _{\underline{p}} \sum _{i=1}^{n} | \underline{z}_{\text {Cole}, i}(\underline{p}) - \underline{z}_{\text {meas},i} |^2, \end{aligned}$$

with *n* frequency points of the measured impedivity $\underline{z}_{\text {meas}}$.

Promising features based on these model parameters were determined with an analysis of variance, where the score of each feature is defined as

5 $$\begin{aligned} p_\textrm{score} = - \log p. \end{aligned}$$

**Fig. 7:**
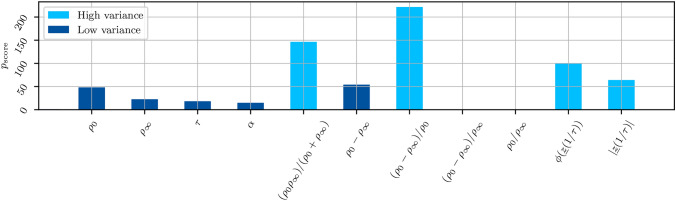
The p-value of all investigated model-based parameter, along with the final choice of features.

The parameter *p* is the *p*-value from a simple ANOVA. A high $p_\textrm{score}$ indicates that the feature has a significant difference between the mean values of the two classes and is suitable for differentiation. According to Fig. 7, the parameters with the highest $p_\textrm{score}$ are

- $\nicefrac {\rho _0\rho _{\infty }}{(\rho _0+\rho _{\infty })}$: an approximation of the intracellular resistivity,
- $\nicefrac {(\rho _{\infty }-\rho _0)}{\rho _0}$: the relative change in resistivity over the spectrum,
- $\phi (\underline{z}(\nicefrac {1}{\tau }))$: the phase of the impedance at the characteristic frequency,
- $|\underline{z}(\nicefrac {1}{\tau })|$: the magnitude of the impedance at the characteristic frequency.

Using these four parameters to represent each measurement results in a feature vector $\varvec{x}_{\text {model}}\in \mathbb {R}^4$ .

## Reference-based feature evaluation

**Fig. 8:**
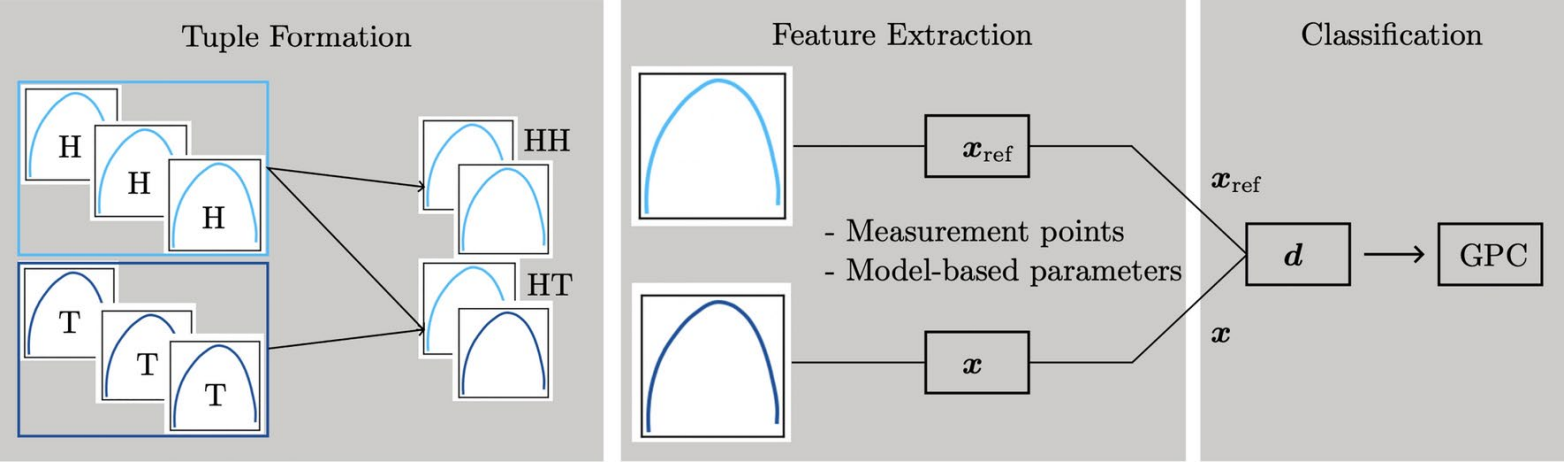
Reference-based classification: measurement tuples are formed per patient, containing either two healthy measurements (HH), or one healthy and one tumorous measurement (HT). For each measurement from the tuple, the feature vector is extracted. The two feature vectors $\varvec{x}$ and $\varvec{x}_\text {ref}$ are related by a distance measure $\varvec{d}$ and form the new feature vector for the GPC.

For each measurement, the two feature vectors $\varvec{x}_{\text {data}}\in \mathbb {R}^{10}$ and $\varvec{x}_{\text {model}}\in \mathbb {R}^4$ are defined. Subsequently, as we want to evaluate the distance of an unknown measurement to a healthy reference of the same patient, adequate measurement tuples are formed. Within these tuples, the distance between the two feature vectors of the same type is determined. The entire process is illustrated in Fig. 8 and will be explained in detail in the following.

### Forming feature tuples

The aim of the GPC will be to classify a unknown measurement knowing the healthy tissue behavior of the same patient. Suitable feature tuples need to be formed - suitable in the sense that one member of the tuple is always healthy, and the other member can be anything. With this approach, the two resulting classes are healthy-healthy (*HH*) and healthy-tumorous (*HT*), i. e. the unknown measurement is healthy or tumorous, whereas the reference is, obviously, always healthy. Pairs of the type tumorous-tumorous do not exist. Hence, on a patient-by-patient basis, each healthy measurement is paired with each other healthy measurement. Then, each healthy measurement is paired with each tumorous measurement, i. e.

6 $$\begin{aligned} H_iH_j =&(\varvec{x}_{\textrm{H}i}, \varvec{x}_{\textrm{H}j}), \end{aligned}$$

7 $$\begin{aligned} H_iT_j =&(\varvec{x}_{\textrm{H}i}, \varvec{x}_{\textrm{T}j}), \end{aligned}$$

where $i\ne j$ and $\varvec{x}$ is the feature vector (either measurement-point-based or model-based). In a first step, all possible tuples are formed. Within the classification step, only a certain amount of tuples per patient will be used in order to keep the dataset balanced.

### Distance evaluation between features in a tuple

Within each tuple, the distance between the two feature vectors has to be quantified. Regardless of the type of features and the vector dimension $n_\text {f}$, each tuple consists of the feature vector $\varvec{x}_\text {ref}= [x_{\text {ref}, 1}, \ldots , x_{\text {ref},n_\text {f}}] \in \mathbb {R}^{n_\text {f}}$ of the healthy reference measurement and the feature vector $\varvec{x}= [x_1, \ldots , x_{n_\text {f}}] \in \mathbb {R}^{n_\text {f}}$ of the measurement to be classified. The distance of these feature vectors is defined via the element-wise difference

8 $$\begin{aligned} \varvec{d}_\text {abs}= [d_{1}, \ldots , d_{n_\text {f}}] \ \text {with} \ d_{i} = |x_{\text {ref}, i} - x_i|. \end{aligned}$$

This metric is chosen because covers both a decrease and an increase in tumor impedance compared to the healthy reference tissue and has proven to yield the best outcome in preliminary studies in comparison to, for example, dividing the feature vectors^76^. Said preliminary results can be found in Appendix B.

### Resulting feature space for different tumor types

**Fig. 9:**
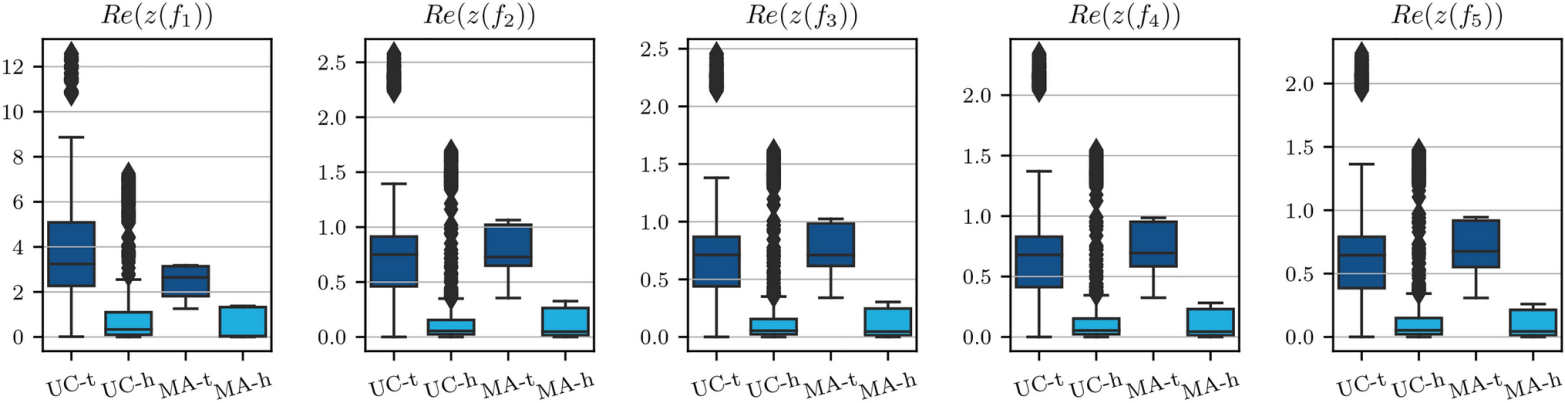
Boxplot for the feature space based on the distance between the real parts of healthy and tumors measurements. The two groups are 1.) tuples from the patients P1-P6 with urothelial carcinoma (UC) and 2) tuples from patient P4 with mucinous adenocarcinoma (MA). The overlap between both groups for the classes “healthy” and “tumorous” indicate that the chosen feature represent impedance characteristics across different tumor types. Note that the UC group has more outliers because of the very large sample size (four patients) in comparison to group MA (one patient).

Before continuing to the classification step, we analyze the feature space some more. With feature space we mean the final input considered for the classifier, hence, the distance within each measurement tuple between the original features defined either based on measurement points or model parameters. Since the data set is very diverse, the question arises if all tumor types can be captured by this same feature space. Especially the mucinous adenocarcinoma (MA) from P4 is histologically different from the urothelial carcinomas (UC). However, our analysis indicates that there is no significant domain gap. As an example, the boxplots of the feature space derived from the real part of the reduced measurement are depicted in Fig. 9. It is obvious that there is a significant overlap between the UC and MA groups. Further, the Mann-Whitney U test, which assesses whether one group has consistently higher or lower feature values, revealed no significant differences. This suggests that the feature space used in our work is not tumor type-specific but instead captures shared electrical properties across the different tumor types. This underlines that the different tumor types are characterized by the same phenomena of proliferation which can be quantified via our reference-based impedance approach.

## Gaussian process classification

After forming the measurement tuples and evaluating the distance within each tuple for both types of proposed features, measurement point-based and model parameter-based ones, the tuples are classified with a GPC. In a first step, two patients are split off from the dataset and will later be used as test data. The remaining data is used to train the GPC in a cross validation fashion. Its success is then evaluated on the unseen test data that was put aside. The special advantage of using a GPC in this context is that is has only two parameters to be optimized and can allow for a good generalization even on our relatively small dataset and serve as a first proof of concept for robust impedance-based tissue differentiation in the bladder.

### Train-test split

The measurements of P1, P2, P3, and P5 are used as training data. To keep the data balanced, i. e. avoiding that some patients are more present than other, exactly 400 randomly selected tuples per class *HH* and *HT* from each of the training patients are used, which leads to $n_\text {s} = 3.200$ samples. Patients P4 and P6 serve as test data to investigate the suitability of the procedure for different scenarios. More specifically, these patients illustrate the two worst case scenarios as they represent the most contradicting measurements in our dataset:

- *P4 - special type of tumor and preoperative therapy*: The tumor has a lower impedance than the healthy tissue, even though literature suggests increased tumor impedance. On top of that, healthy tissue impedance is strongly increased in comparison to literature data^74^.
- *P6 - untherapized, highly undifferentiated urothelial carcinoma with tremor influences*: This is the most common tumor type where the tumor has a higher impedance than the healthy tissue. In addition, the measurements are recorded with the handheld sensor and suffer from a unsteady sensor-tissue contact.

### Cross-validation procedure

**Fig. 10:**
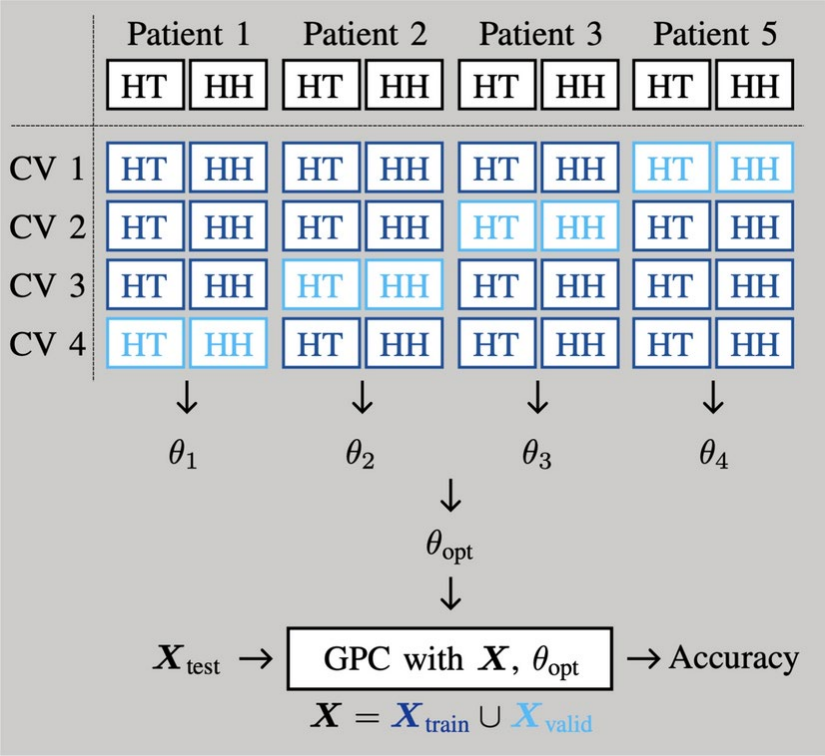
Cross validation scheme for the training of the GPC. For each step, three patients are used for training, one patient for validation. The final parameters $\theta _\text {opt}$ are determined based on the training steps with high validation accuracy.

A 4-fold cross-validation, illustrated in Fig. 10, is undertaken to train the GPC and determine optimal parameters. For each fold, the training data $\varvec{X}$ is divided into four subsets, where three patients are used for training and one patient serves for validation. With this methodology, overfitting or learning of specific patient data is avoided.

*Cross-validation folds* In the *i*-th cross-validation fold, $i \in \{1,\ldots ,4\}$, scaling functions based on the respective training data $\varvec{X}_{\text {train},i}$ are determined and both training and validation data are scaled to zero mean and unit variance. A binary GPC with latent function $f_i~\sim GP(\varvec{0}, k(\varvec{d}, \varvec{d}'))$, kernel $k(\varvec{d}, \varvec{d}')$ and logistic activation function is used for classification. The kernel specifies the covariance between the random variables and thus enables the description of the covariance $\text {cov}(f\left( \varvec{d}),f(\varvec{d}')\right)$ between the outputs on the basis of the covariance $k(\varvec{d},\varvec{d}')$ between the inputs. The use of a kernel does not require an explicit specification of basis functions and theoretically allows for infinite dimensional feature spaces. This makes the problem formulation less complex and reduces the computational effort. A standard kernel for classification tasks is the stationary radial basis function kernel^77^, which, in this work, is based on the distance measure $\varvec{d}$ to a healthy reference measurement, i. e.

9 $$\begin{aligned} k(\varvec{d}, \varvec{d}') = \sigma _\text {f}^2 \exp \left( -\frac{\Vert \varvec{d} -\varvec{d}'\Vert ^2}{2l^2}\right) , \end{aligned}$$

with the parameters signal variance $\sigma _{\text {f}}^2>0$ and length scale $l>0$. The initial conditions of these parameters are set to $\sigma _{\text {f},0}=1$ and $l_0=1$.

In each cross-validation step, the GPC is trained on the training subset $\varvec{X}_{\text {train},i}$ in the sense that the optimized kernel parameters

10 $$\begin{aligned} \varvec{\theta }_i=[\sigma _{\text {f},i}^2, l_i] \end{aligned}$$

maximize the logarithm of the marginal likelihood. The process is implemented with *scikit-learn*^78^ and we refer to^77^ for additional information on Gaussian process classification. The performance of the GPC in each cross-validation step is evaluated based on the classification accuracy achieved on the validation subset $\varvec{X}_{\text {valid},i}$. If the validation accuracy is higher than the threshold $c_\text {acc}=0.8$, the parameters $\varvec{\theta }_{\text {opt},i}$ are included in a set

11 $$\begin{aligned} \Theta =\left\{ \varvec{\theta }_i \ | \ \text {validation accuracy} \ge c_\text {acc} \right\} . \end{aligned}$$

**Fig. 11:**
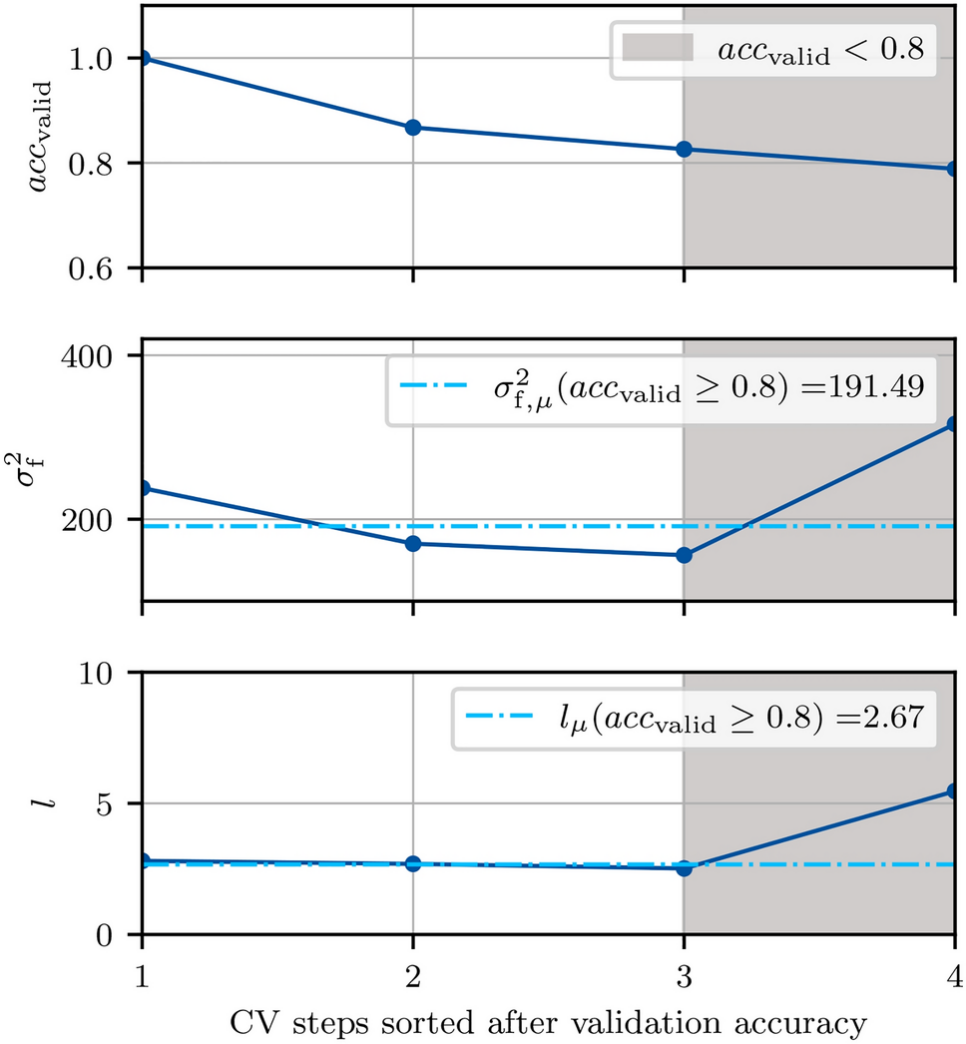
Model selection on the basis of four cross-validation steps which have already been sorted according to validation accuracy. From the steps with a validation accuracy above the defined threshold of 80%, the kernel parameters $\sigma _\text {f}$ and *l* are added to the set $\theta$, whose mean values are used for the kernel of the final GPC model.

The training results in terms of length scale, variance, and resulting validation accuracy are shown in Fig. 11. In three out of four steps, a classification accuracy above the threshold is achieved. For these three steps, the both the length scale *l* and the signal variance $\sigma _\text {f}^2$ are very similar.

*Model selection* After iterating over all four combinations of the training and validation splits, the set $\Theta$ contains the optimized parameter tuples that yield a high classification accuracy on the validation sets. Taking their mean values yields the optimized parameters $\varvec{\theta }_\text {opt}=[\sigma _{\text {f,opt}}^2, l_\text {opt}]$, and, thus, the final kernel

12 $$\begin{aligned} k_\text {opt}(\varvec{d}, \varvec{d}') = \sigma _\text {f,opt}^2 \exp \left( -\frac{\Vert \varvec{d} -\varvec{d}'\Vert ^2}{2l_\text {opt}^2}\right) . \end{aligned}$$

Within our training process as depicted in Fig. 11, we obtain $\sigma _{\text {f,opt}}^2=191.49$ and $l_\text {opt}=2.67$. To define the final model, a GPC is given the entirety of the training set $\varvec{X}_\text {train}$ with the fixed kernel $k_\text {opt}(\varvec{d}, \varvec{d}')$ to have the most amount of prior information possible.

### Classification results

**Fig. 12:**
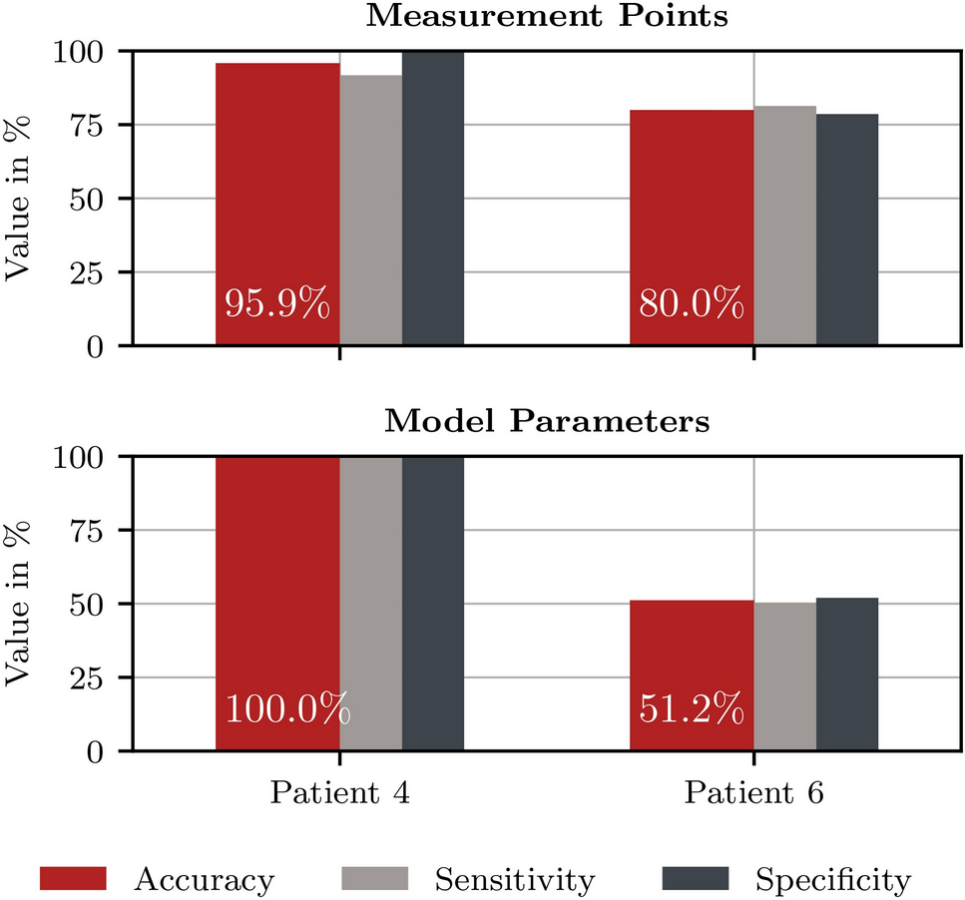
Classification accuracy (), sensitivity (), and specificity (). The results when using raw measurement points of five selected frequencies as feature vector $\varvec{x}_\text {data}$ are depicted in the top plot. The results with model-based parameters as feature vector $\varvec{x}_\text {model}$ are depicted in the bottom plot.

**Fig. 13:**
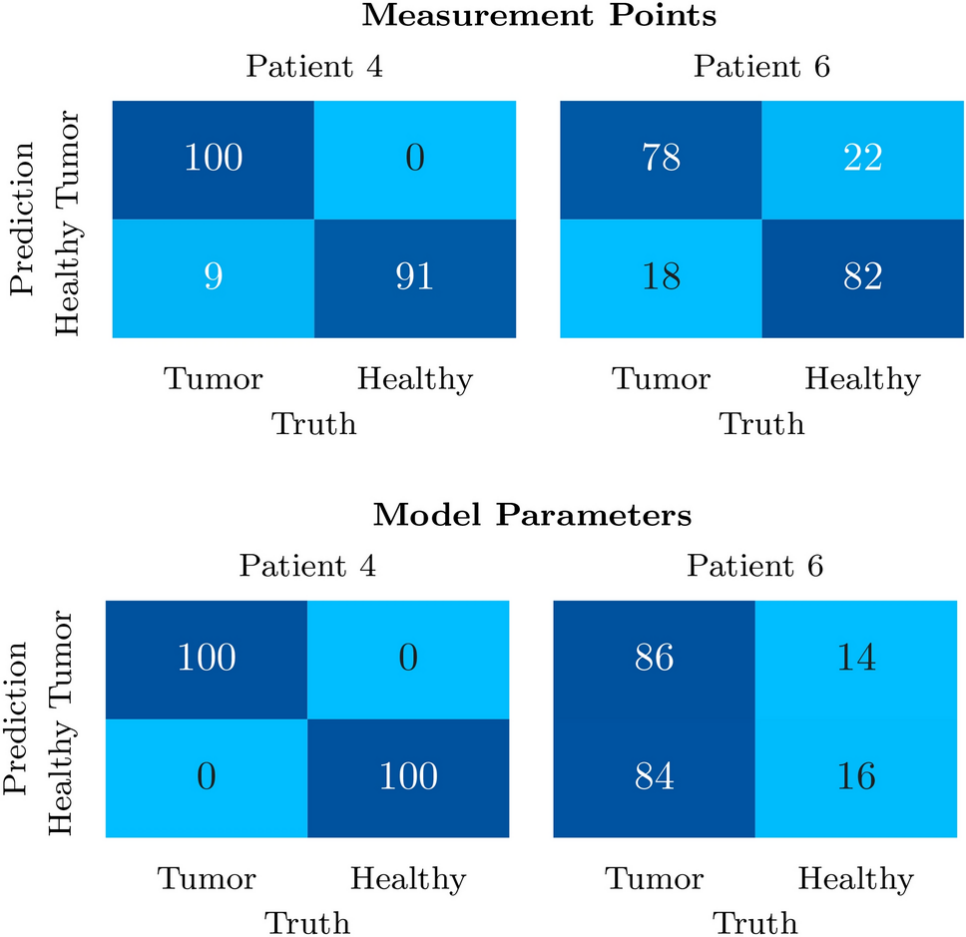
Confusion matrices of the test data from P4 and P6 for both measurement point features and model parameter features. Upper left: true positives. Upper right: false positives. Lower left: false negatives. Lower right: true negatives.

The performance of using measurement points and model-based parameters as feature vectors are evaluated separately based on the balanced accuracy as well as the sensitivity (true positive rate) and specificity (true negative rate)

13 $$\begin{aligned} \text {sensitivity}&= \frac{\text {TP}}{\text {TP}+\text {FN}} \end{aligned}$$

14 $$\begin{aligned} \text {specificity}&= \frac{\text {TN}}{\text {TN}+\text {FP}} \end{aligned}$$

15 $$\begin{aligned} \text {balanced accuracy}&= \frac{\text {sensitivity + specificity}}{2} \end{aligned}$$

with true positive (TP), false positive (FP), true negative (TP), and false negative (FN). The balanced classification accuracy, sensitivity and specificity of the test data $\varvec{X}_\text {test}$ (P4 and P6) obtained with said final model is shown in Fig. 12, along with the confusion matrices in Fig. 13. For comparison, in our previous classification approach, where only structurally similar measurements without external disturbances were considered and no reference was included, we achieved a classification accuracy of 91.7% with NNs and 88.4% with SVMs for P4^54^.

*Patient P4* With the approach followed in this work, patient P4 shows a even higher classification accuracy of 97.75% on average for both feature types, with the results of the individual features and distance types being 95.5% for measurement point features and 100% for model-based features. This outcome exceeds the test accuracy of the classification from our earlier work^54^. While specificity and sensitivity are both 100% for model-based features, measurement points also obtain a specificity 100%, but sensitivity decreases to 91.7%.

*Patient P6* Even when the differentiation is more ambiguous due to external disturbances and a visual differentiation of the measurements is nearly impossible, the presented approach still achieves a classification accuracy of 80% for features based on a reduced measurement vector. The performance is significantly worse for model-based features which only achieve a classification accuracy of 51.2% with similarly low sensitivity and specificity. Note that the P6 test data is not balanced and consists of 120 *HH* and 224 *HT* tuples, which is why we used the balanced accuracy as evaluation metric.

## Discussion

As already mentioned, our earlier work dealt with investigating classification approaches of impedance data taken with controlled and uniform sensor-tissue contact force. Features based on physical model parameters such as the Cole model resulted in better classification accuracy than using measurement points.

*Challenging parameter identification* However, we realized quickly that, once the impedance measurement is distorted or not smooth, the extraction of said physical parameters becomes challenging as the real data often differs too much from the model assumptions. In our specific case, this means that the measurement is not represented as a half circle in the complex plane anymore - even though the fitting error of the least squares model fit was still within a reasonable range. Hence, even with relatively small fitting error, it is possible that the Cole parameters will not represent the measurement accurately. This is exactly the reason why a model-based classification of P6 results in a worse classification accuracy. Looking at Fig. 13, it is particularly interesting that the model-based features have a very high false negative rate and a low false positive rate. This underlines that the distance between tumorous and healthy model parameters is just not enough to allow for a correct classification with these measurements. Although using model parameters provides an approach to interpreting biological processes, it should be used with caution, especially in the context of clinical application. Biological tissue is highly complex and even small external perturbations or preoperative therapy significantly affect the measured behavior. We acknowledge that a physics-based model that represents different domains of the tissue can definitely offer added value. However, especially in the intraoperative situation, a purely model-based evaluation of the impedance measurement for classification, without patient-specific prior knowledge, can lead to misinterpretation, especially with challenging measurement conditions or preoperative therapy involved. For this application, physics-informed machine learning models might offer added value and should be investigated in the future.

*Robust classification with measurement points* For this reason, the classification approach based on measurement points is more suitable for a reliable intraoperative tissue differentiation. Especially with regard to the very small distance between the tumorous and healthy measurements of P6, the result of 79.9% is to be evaluated as a very first promising step towards robust intraoperative tissue classification for different tumor types and therapies involved. Furthermore, it is to mention that the incorporation of the healthy reference further improves the classification of P4, which had already achieved high classification accuracies in our earlier work, even when using the slightly worse performing measurement point features. However, the measurement point-based approach still results in a specificity of 100%: high specificity is important because tumorous tissue that is accidentally classified as healthy tissue is most dangerous case for the clinical application. A high specificity (true negative rate) is essential.

*Time saving aspect* We want to underline again that the result with the measurement feature vector only consist of *five selected frequencies*: Using five selected frequencies reduces measurement time by 85% in comparison to the model-based approach, where a sufficient number of measurement points is essential for the model identification, i. e. 7s for a sweep with 50 frequencies vs. 1 s for a sweep with five frequencies with the impedance analyzer used in this work. Additionally, no further post processing is involved.

*Limitations of this work* While this study demonstrates the potential of electrical impedance measurements to differentiate between healthy and tumorous bladder tissue, several limitations must be acknowledged: The dataset size and diversity may not fully represent the variability in real-world conditions. Future studies should include more samples to improve generalizability. Additionally, variations in tissue preparation and measurement conditions can introduce noise, needing further standardization, especially in terms of contact force between sensor and tissue. Last but not least, tumor heterogeneity and the effects of margins between healthy and tumorous tissues may pose challenges in clinical settings. Future work should investigate these effects and validate the approach against gold-standard diagnostic methods.

## Conclusion

Over the years of research, it became obvious that electrical impedance spectroscopy is a very promising tool for tissue differentiation, but also a very sensitive one. This work presented an impedance-based bladder tissue classification concept able to deal with the general problem that wide variations in impedance values occur, which are mainly due to external disturbances, such as deformation, preoperative radiotherapy, systematic measurement error, different tumor types within one bladder, or just inter-individual differences in age, gender, health. To deal with the challenges, we present a reference-based approach, aiming to allow for a *relative* classification of the measurement in comparison to a healthy reference, making it suitable to be applied in endoscopic surgery scenarios. More specifically, we evaluate the euclidian distance of the features of the unclassified measurements to the healthy reference features obtained from the same patient. While a classification accuracy of up to 100% is achieved on unseen, noise-free test data, classification accuracy on ambiguous measurements is only acceptable when using selected measurement points as a purely (physics-) model-based analysis does not always truly represent tissue properties. However, the classification only based on the impedance of five selected frequencies reaching an accuracy of 80%, shows the advantage of the method but also underlines that there is still research to be done.

The takeaway message of this work is that a certain distance between the impedance measurements of the different pathological conditions within the patient is necessary for a successful classification. Distance in this sense is defined as the subtraction of selected features of the measurement from healthy reference values of the same patient. To ensure a clear differentiation, controlled testing conditions for the impedance measurements are crucial and need to be considered in probe construction for future in vivo applications.

## Supplementary Information

Supplementary Information.

## Data Availability

The dataset generated and analysed during the current study is available in the github repository https://github.com/cayobro/electrical-impedance-data-bladder.git.

## Footnotes

1 This research is approved by the ethics committee of the University Hospital Tübingen and the Eberhard Karls University Tübingen under grant 538/2021BO1. The patient material is used in this study after obtaining informed consent from the donor in writing.
