# Supplementary material for: Electrical impedance-based tissue classification for bladder tumor differentiation
Source: Sci Rep. 2025 Jan 4;15:825. doi: 10.1038/s41598-024-84844-9 (PMC11700109; doi:10.1038/s41598-024-84844-9)
Supplement: Supplementary file 1 — Supplementary Information. [file 41598_2024_84844_MOESM1_ESM.pdf]

Appendix A Histology Reports

The primary tumor types, their grading, and therapies are specified in Table S1.

Table S1 Histology reports from the bladders used.

Patient	Primary tumor type
1	Neoadjuvant treated sarcomatoid urothelial carcinoma, grading: not applicable, TNM: type4a, TNM: Typ4a
2	Urothelial carcinoma of the urinary bladder, grading: not applicable after neoadjuvant therapy. TNM: type3b
3	Urothelial carcinoma of the urinary bladder with squamous differentiation, grading: G3, TNM: pT2b(is)
4	Mucinous adenocarcinoma of the urachus, grading: G3, TNM: pT3b
5	Muscle-invasive urothelial carcinoma of the urinary bladder, grading: G3, TNM: pT2b
6	Urothelial carcinoma of the urinary bladder, grading: high-grade/G3, TNM: pT4a (is)

Appendix B Distance Measures between Features

For the feature evaluation, we define the absolute distance $\boldsymbol{d}_{\mathrm{abs}}$ between two features. Other distance measures include the scaling of this difference by the respective maximum value of the feature,

$$\boldsymbol{d}_{\mathrm{norm}} = [d_{\mathrm{norm},1}, ..., d_{\mathrm{norm},n}] \text{ with } d_{\mathrm{norm},i} = \frac{|x_{\mathrm{ref},i} - x_i|}{\max(x_{\mathrm{ref},i}, x_i)}; \tag{B1}$$

or the division of the features,

$$\boldsymbol{d}_{\mathrm{div}} = [d_{\mathrm{div},1}, ..., d_{\mathrm{div},n}] \text{ with } d_{\mathrm{div},i} = \frac{\min(x_{\mathrm{ref},i}, x_i)}{\max(x_{\mathrm{ref},i}, x_i)}, \tag{B2}$$

where the numerator is always defined over the maximum value of both features. The normalization, or division, by the larger feature in each case, is important to cover impedance changes in both directions. The classification results of all distance measures are depicted in Figure S1 for the sake of completeness. The training and test procedure was equivalent to the what was presented in the main manuscript. Whereas all distance measures were successful for the data of P4, only the approach with $\boldsymbol{d}_{\mathrm{abs}}$ worked for the unsteady measurements of P6 and was pursued in the scope of this work.

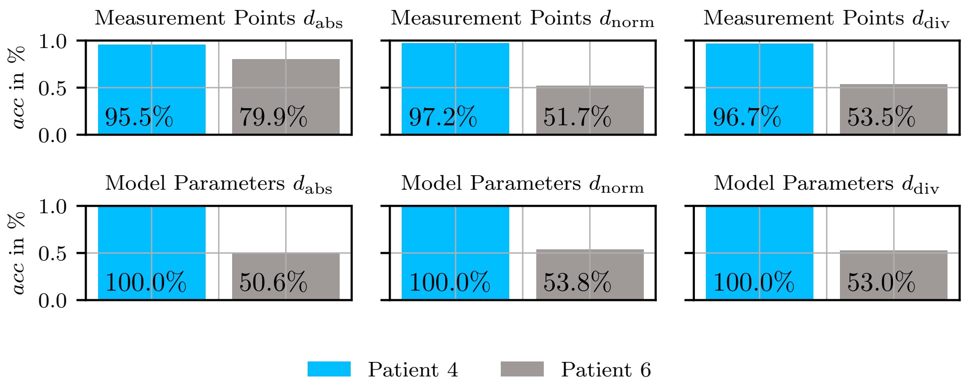

Fig. S1 Entirety of classification results for different distance measures.
